# Thermochemical upcycling of paper-mill waste into hydrocarbon-rich fuels and functional char for rubber reinforcement

**DOI:** 10.1039/d5ra08655h

**Published:** 2026-02-23

**Authors:** Mukesh Bhatt, Madhu Ganesh, Ranjeet Kumar Mishra, Abhishek Sharma, Jyeshtharaj B. Joshi

**Affiliations:** a Department of Biotechnology and Chemical Engineering, Manipal University Jaipur Rajasthan 303007 India abhishek.sharma@jaipur.manipal.edu; b Department of Aerospace Engineering, Karunya Institute of Technology and Sciences Coimbatore 641114 Tamil Nadu India; c Manipal Institute of Technology, Manipal Academy of Higher Education Manipal India ranjeet.mishra@manipal.edu; d Department of Chemical Engineering, Institute of Chemical Technology Mumbai-400019 Maharashtra India; e Department of Chemical Engineering, Birla Institute of Technology and Science, Pilani K K Birla Goa Campus Zuarinagar Sancoale Goa 403726 India

## Abstract

This study investigates the thermochemical upcycling of paper-mill waste composed of mixed paper and residual plastics into high-value fuels and functional char for composite applications. Co-pyrolysis was conducted in a semi-pilot-scale rotary kiln reactor at 450–650 °C, producing maximal pyrolysis oil yields of 48 wt% and 20 wt% char at 650 °C. The pyrolysis oil was separated into heavy and light fractions, with calorific values of 39–43 MJ kg^−1^, demonstrating strong fuel potential comparable to that of petroleum diesel. GC-MS analysis revealed the presence of hydrocarbons, alcohols, and aromatic compounds, including toluene, styrene, and xylene, indicating synergistic degradation of cellulosic fibres and polyolefin plastics. Furthermore, vacuum distillation improved oil quality by enriching the C_6_–C_18_ hydrocarbon fractions and reducing the oxygenated species. The char exhibited a high carbon content (71.20%), a moderate ash content (30.30%), and an energy value of 16.5 MJ kg^−1^, along with a porous morphology and mineral traces, enabling its use as a composite filler and secondary fuel material. Rubber compounding trials using SBR-1502 demonstrated that replacing carbon black with pyrolytic char yielded acceptable performance: hardness (58 Shore A) and tensile strength (18 kg cm^−2^) were lower than the standard carbon-black compound (63–64 Shore A; 123 kg cm^−2^), yet significantly higher than the filler-free control. Notably, char-reinforced rubber displayed similar elongation (322%) and a reduced curing time (*T*_c90_ = 9.1 min), highlighting faster vulcanisation due to catalytic ash components. These results confirm the potential of paper-mill waste pyrolysis for sustainable fuel production and the recovery of circular-economy materials.

## Abstract 2

This study investigates the thermochemical upcycling of paper-mill waste composed of mixed paper and residual plastics into high-value fuels and functional char for composite applications.

## Graphical abstract

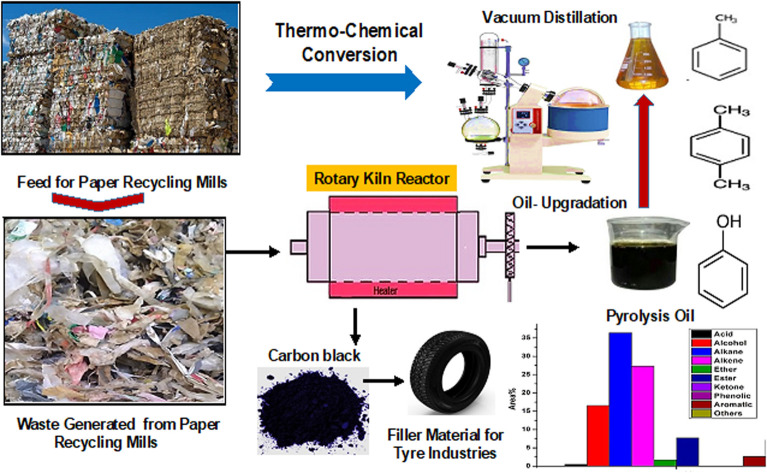

## 1 Introduction

The trend towards sustainability in the pulp and paper industry involves recycling waste paper. In India, approximately 85% of paper mills use imported wastepaper as the primary fibre source for the production of paperboard, newsprint, and paper. India generates approximately 15 million tonnes of paper and paperboard from the paper industry, and generates massive amounts of plastic waste (3% of total production), creating disposal challenges.^1,2^ The Indian paper industry has an average growth rate of around 6%, compared to the global average of approximately 2.4% in paper and paperboard production. The industry is highly fragmented, consisting mainly of small and medium-sized paper mills located across the country.^3^ In India, the majority of paper mills are located in clusters, primarily in Gujarat, Tamil Nadu, Uttar Pradesh, Maharashtra, and Punjab, which together account for around 50% of total paper and board production. On the other hand, globally, despite high recycling rates, the U.S., faces the ongoing challenge of paper waste in landfills, constituting about 28% of landfill mass.^4^ As the world's largest importer of waste paper, handling approximately 20 million tons annually, China faced challenges in scientifically treating the waste generated from paper recycling mills. Consequently, China implemented a ban on waste paper imports to address these environmental concerns. European countries, led by regulations like the EU's Industrial Emissions Directive, focus on the Best Available Techniques (BAT) to treat the waste from paper recycling waste.^5^ Over the years, the global hub for importing waste paper has undergone significant shifts. Initially centred in Europe, it later moved to East Asia and now finds its home in Southeast Asia. Despite this shift, Asian countries continue to grapple with a significant challenge: managing the waste produced by paper recycling mills.^6^

The majority of paper manufacturing units produce a very similar range of paper and board products, including unbleached packaging grades. Plastic waste is a by-product of the initial paper recycling process. Segregation of waste plastic is difficult as it is mixed with short paper fibers, pins, stones, denim clothes, cotton, fillers, ink particles, metals, and non-metals.^7^ Conventional methods of disposing of waste plastic include landfilling and incineration. Paper mill waste plastics could be mitigated using emerging techniques and methodologies, depending on the type of plastics and associated impurities.^8^ Thermal treatment methods, such as combustion, pyrolysis, and gasification, have been extensively researched as alternative approaches for valorising solid organic waste materials, including polymers, waste paper, and rubber. Gasification involves a high-temperature (>900 °C) partial oxidation to produce flammable gases. Pyrolysis is a method of thermally decomposing a raw material in the absence of oxygen into a solid residue (char) and volatile fractions. Pyrolysis operates at lower temperatures (400–900 °C) than gasification, which can help preserve the chemical composition of the feedstock and minimize undesired side reactions, resulting in higher-quality products.^9,10^

The yield of pyrolysis liquid from the co-pyrolysis of mixed feedstock depends on the process parameters and the quality of the feedstock. In most studies, plastic has been recognised as a good source of liquid fuel. Plastic pyrolysis has led to a significant increase in pyrolysis oil yield, particularly with polystyrene.^11,12^ Pyrolysis oil primarily consists of aromatics and aliphatic hydrocarbons, along with other oxygenated compounds.^13^ Alkanes (methane, ethane, heptane, octane, propane), alkadienes and alkenes are mainly present in aliphatic hydrocarbons. Polycyclic aromatics (naphthalene, methylnaphthalene, and ethyl naphthalene) and aromatic hydrocarbons include styrene, toluene, benzene, cumene, ethyl benzene, and monocyclic aromatics (alkyl benzenes, alkyl toluene). On the other hand, pyrolytic oil from biomass contains 15–50 wt% water and 35–40 wt% oxygen, resulting in low-calorific fuel.^14^ Generally, the HHV of pyrolysis oil from biomass is about 15–26 MJ kg^−1^. In comparison, the pyrolysis oil produced in plastics has an HHV of 29–43 MJ kg^−1^.^15^ To understand product quality, studying the co-pyrolysis of waste paper with mixed plastics is important. Ali *et al.* (2005) carried out pyrolysis of waste streams from paper mills that recycle paper, containing significant amounts of plastic in pellet form, at 300 °C, 425 °C, and 550 °C using a fixed-bed reactor. At 425 °C, char and heavy pyrolysis oil phase products with HHVs of up to 33 MJ kg^−1^ and 42.80 MJ kg^−1^, respectively, were produced. Thermal conversion of different waste streams containing varying amounts of plastics with waste paper pulp decreased at 550 °C but favoured the production of the heavy pyrolysis oil phase, thereby decreasing the quality of the char in terms of fuel value (HHV < 21 MJ kg^−1^).^16^ In another study, the co-pyrolysis of waste newspaper and high-density polyethylene with different weight ratios at 500–800 °C in the pyroprobe was carried out. At 600 °C, with an equal weight ratio of feedstocks, pyrolysis yielded its maximum pyrolysis oil, containing 85.88% alcohols and hydrocarbons. Hydrogen transfer and deoxygenation by polymer and recombination reactions increased the amount of branched hydrocarbons. Radicals from degradation favoured secondary cracking of HDPE products, leading to the formation of linear hydrocarbons with low carbon numbers. Moreover, the quality of condensable pyrolysis oil was upgraded *via* co-pyrolysis.^17^ Furthermore, the co-pyrolysis study indicated that mixing plastics with biomass could significantly impact the quality and composition of the pyrolysis products. The formation of hydrocarbons was also enhanced in the co-pyrolysis of stem wood, plastics, and paper rejects. Co-pyrolysis also affected the properties of pyrolysis oil, including higher viscosity and acidity. Oxygenated compounds, such as acids, aldehydes, and ketones, decreased, while more stable compounds, such as esters and alcohols, increased.^18^ In recent years, biochar has become a sustainable material of interest as filler or composite material, as a substitute for carbon black. Biochar is generally rich in carbon and can be produced by pyrolysis of agricultural or industrial solid wastes.^19^ It was observed from literature studies that the physical and chemical characteristics of the feedstock, pyrolytic conditions, along with the mixing ratio (char: carbon black), and the carbon content of the pyrolysis char, affect the mechanical and rheological properties of composite materials.^20–23^ After reviewing several research articles, it was found that few studies have investigated the potential of the pyrolytic process to convert waste generated by paper recycling industries into high-grade pyrolysis oil as a fuel and secondary products.^24,25^ Further, to the best of the author's knowledge, no study has been conducted to investigate, at a larger scale, the quality of products obtained from the thermochemical conversion of residual plastic waste from paper recycling industries and the upgradation of pyrolysis oil. Particular attention has been directed toward the fuel properties of the pyrolysis oil and the suitability of the resulting char as a filler material.

In light of the aforementioned research gap, this study investigated the co-pyrolysis of paper waste and plastics to produce renewable pyrolysis oil. The experiment was performed in a rotary kiln reactor at temperatures between 450 and 650 °C to produce pyrolysis oil. The pyrolysis oil obtained from thermochemical conversion has been upgraded through vacuum distillation, and its properties, including calorific value, water content, and other fuel properties, have been discussed in detail. These properties have been compared with those of diesel fuel. Furthermore, the solid residue (char) obtained from the test was analysed using XRF, SEM, and EDX, and its rubber compounding properties were tested, including tensile modulus, elongation, and strength. Additionally, carbon black was used to assess its suitability for the rubber and tyre industries.

## 2 Materials and methods

### 2.1. Sample collection and preparation

Polypropylene (PP), waste paper (WP), low-density polyethylene (LDPE), and residual paper-plastic (RWP) wastes were collected from the landfill site in Jaipur, Rajasthan, India. Paper mill waste was collected from Saharanpur Paper & Board Mills, Uttar Pradesh. The waste plastics (PP, LDPE) were washed with hot water to clean impurities, such as dust. Further, waste was dried in an open atmosphere (2–3 days) and placed in a hot air oven at 105 °C for 1 h for uniform removal of moisture content. The oven-dried waste was pulverized into different shapes and sizes (50–80 mm) and placed in an air-tight plastic container to prevent moisture absorption.

### 2.2. Physicochemical characterisation of feeds

The proximate and ultimate analyses of feedstocks are essential for assessing their bioenergy potential. The proximate analysis was conducted following ASTM D3173, ASTM D3174, and ASTM D3175 standards, while the ultimate analysis was performed using a CHNS elemental analyser (Variael CUBE, Germany). Further, the higher heating value (HHV) of the feeds, pyrolysis oil, and char was calculated using the Dulong formula (eqn (1)), based on the elemental composition of the samples.^26^ The bulk density of the feeds was determined using a digital balance and a graduated cylinder. The sample's weight was measured using a digital balance, and its volume was measured using a graduated cylinder.

1 HHV (Mj kg)^−1^=327934 + 0.5321C^2^ − 0.5321C − 2.8769H + 0.608CH − 0.2401N

where, HHV = higher heating value, C = carbon, H = hydrogen, N = nitrogen.

### 2.3. Thermal stability analysis

Thermal analysis of feeds was conducted using a TGA (NETZSCH, TG 209 F1 Libra) in an inert atmosphere. Approximately 9.0 mg of the sample was placed in a crucible for each experiment. The heating process started at 25 °C and increased to 900 °C at a rate of 10 °C min^−1^, with an inert gas (nitrogen) flow rate of 40 mL min^−1^.

### 2.4. FTIR and XRF analysis

The presence of functional groups in the feeds was analysed using FTIR (FTS-3500GX with DRS). The dried KBr powder was uniformly mixed with the feed at a 1 : 100 ratio and placed in the sample holder. Further, the scanning was performed at a rate of 40 with a step size of 4 cm^−1^, covering a wavelength range of 400–4000 cm^−1^. The inorganic elemental composition of biochar was analysed using X-ray fluorescence (XRF) spectroscopy (Epsilon-1 Panalytical, Germany). Biochar samples were combusted to obtain ash, which was then finely ground to ensure homogeneity. The instrument irradiated samples with primary X-rays, causing the atoms to emit characteristic fluorescent X-rays that were used to identify and quantify the elements. Major and trace elements, including Na, Al, K, Ca, Si, Fe, P, and Mg, were detected. All analyses were conducted under standardised, factory-calibrated conditions to ensure measurement accuracy, precision, and reproducibility.

### 2.5. Experimental setup

**Fig. 1:**
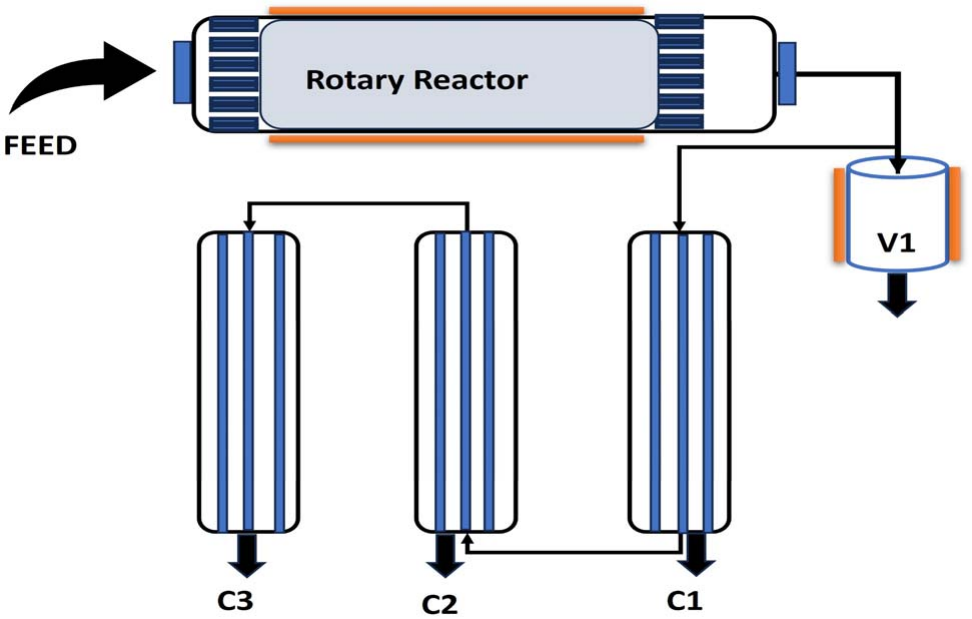
Systematic experimental layout of the Rotary kiln pyrolysis reactor system.

In this study, a semi-batch rotary kiln reactor was utilised for the pyrolysis of feedstocks, as illustrated in Fig. 1. The reactor consisted of a horizontal stainless steel tube measuring 1360 mm in length, with an outer diameter of 355 mm and a wall thickness of 3 mm. The rotary kiln drum was connected to a primary oil collection vessel, followed by three condensers in series. Additionally, three oil collection vessels were attached to the bottom of the condensers. A gas flowmeter (Raychem G1.6 BS EN 1359) and a compressed knitted stainless steel mesh scrubber were installed at the end of the vapour pipeline. The rotary kiln drum was externally heated by three 9 kW heaters. Additionally, the primary collection vessel's temperature was controlled using a 2 kW electrical heater. For each experiment in the rotary kiln reactor, a 2 kg feed sample was preheated at 150 °C for 1 h to eliminate moisture. Subsequently, the temperature was ramped up to 450 °C, 550 °C, and 650 °C at a rate of 20 °C min^−1^, maintaining each temperature for 90 min while the rotary drum rotated at 1 round per min. Vapours resulting from the devolatilization of the feed material were condensed in a series of condensers. The rotary kiln offers better heat transfer, a homogeneous temperature distribution, and the ability to treat heterogeneous mixtures of paper and plastic without high pre-treatment, compared to fixed-bed reactors, which require 60–120 min of residence time, and fluidised-bed systems, which are unable to process fibrous paper materials, leading to bed agglomeration. The non-catalytic rotary kiln method has operational simplicity and cost-efficiency to industrial-scale operation compared to catalytic pyrolysis methods, which have high sensitivity to ash (high content in our feedstock 5.4%) and need to be re-established with fresh catalysts regularly. After pyrolysis was complete, the char was collected from the rotary drum once it naturally cooled to room temperature. The system underwent thorough cleaning after each experiment to prevent any buildup in the pipelines. The solid, liquid, and gas yields were determined using eqn (2), (3), and (4), respectively.2
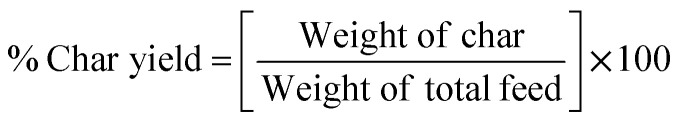
3
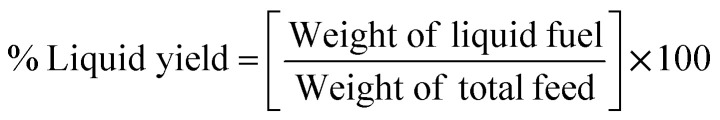

4 % Gas yield = 100 − (% liquid yield + % char yield)

### 2.6. Characterisation of pyrolysis oil

The extraction of chemical compounds from oil based on polarity was performed using hexane, acetone, and benzene. The pyrolysis oil (10 mL) was first mixed with 20 mL of hexane in a separating funnel. The hexane-immiscible layer, which included the polar phase and some insoluble material, was then separated. This fraction was dissolved in 20 mL of acetone, and the acetone-insoluble compounds were further extracted using 10 mL of benzene. The collected pyrolysis oil samples were analysed using a Shimadzu GC-MS QP2020 to determine their chemical composition. The GC-MS was equipped with a 30 m capillary column, and the method involved increasing the oven temperature from 50 °C to 270 °C, using helium as the carrier gas at a flow rate of 2 mL min^−1^. Before analysis, the oil samples underwent pre-treatment: 400 mg of pyrolysis oil was dissolved in 10 mL of acetone. Before injection, the diluted samples were dehydrated with 2 g of anhydrous sodium sulfate and then filtered through a 0.22 µm syringe filter. Elemental analysis was performed using a LECO-Truspec CHNS analyser. Proximate and ultimate analyses were conducted in accordance with the method in section 2.2. An Eutech Ion 2700 was used to measure pH with demineralised water. Viscosity was measured at 40 °C using calibrated glass capillary viscometers (Cannon-Fenske type, Schott Instruments) in accordance with IS 1448 Part 25. The flow time between graduations was recorded and multiplied by the viscometer constant to obtain the kinematic viscosity in centistokes. Density was determined at 15 °C using a digital densitometer (Anton Paar DMA 4500M) in accordance with IS 1448 Part 16, with temperature corrections applied. The flash point was determined using a Pensky–Martens closed cup apparatus (Koehler K16270) in accordance with IS 1448 Part 21. The sample was heated with stirring, and an ignition source was applied at incremental temperatures to identify the flash point. Water content was analysed using Karl Fischer titration (Metrohm 870 KF Titrino Plus) in accordance with IS 1448 Part 40. The sample was dissolved in anhydrous methanol and titrated with Karl Fischer reagent to determine the water content through coulometric or volumetric measurement.

The higher heating value (HHV) was determined using a bomb calorimeter (IKA C200) in accordance with JL/STP/015. The dried sample was combusted under controlled oxygen pressure, and the temperature rise in the water bath was measured and calibrated against benzoic acid standards to calculate energy content in MJ kg^−1^. Sulfur content was analysed per IS 1448 Part 33 by oxidising the sample and quantifying sulfate using X-ray fluorescence spectroscopy (Rigaku ZSX Primus II) with standard calibration. Copper strip corrosion was tested in accordance with IS 1448 Part 15 by immersing polished copper strips in the sample at 100 °C for 3 h, followed by visual examination and rating against ASTM standards. Sulphate ash was determined in accordance with IS 1448 Part 4 by igniting the sample in a muffle furnace at 775 °C, treating it with sulfuric acid, re-igniting, and weighing the residue to calculate the percentage. Total contamination was measured per IP 440 by vacuum-filtering the sample through pre-weighed 0.8-micron membrane filters, then drying at 105 °C and weighing the particles to determine contamination in mg kg^−1^. Carbon residue was evaluated per IS 1448 Part 8 by heating the sample in an open crucible until complete decomposition, then weighing the carbonaceous residue. Inorganic acidity was determined in accordance with IS 1448 Part 2 by extracting with isopropyl alcohol and titrating with a 0.1 M TBAOH solution. A combination pH electrode with an Ag/AgCl reference (Metrohm 6.0259.100) was used to detect the endpoint and quantify carboxylic acids and phenolics. Sediment content was analysed according to IS 1448 Part 30 by centrifuging and vacuum-filtering to isolate the sediments, which were then dried and weighed as a percentage of the sample mass.

### 2.7. Characterisation of char

The proximate and elemental analyses of the char were conducted using the procedures and methods outlined in Section 2.6. Energy-dispersive X-ray spectroscopy (EDX) was employed to estimate the mineral content in the raw biomass. A FESEM/EDX analyser (ZEISS, Sigma) was used to determine the percentage of mineral matter. A small amount of dried biomass was placed on carbon tape and coated with gold to prevent charging of the sample during analysis.

## 3 Results and discussions

### 3.1. Physicochemical characterization of feeds

The physicochemical characterisation of polypropylene (PP), waste paper (WP), low-density polyethylene (LDPE), and residual paper-plastic (RWP) is listed in Table 1 and compared with other reported studies, such as polypropylene,^27^ low-density polyethylene,^28^ waste office paper,^29^ and waste nitrile gloves.^30^ Residual paper plastics contain mainly a mixture of LDPE, PP, and plastic-coated paper fibres. From Table 1, it was observed that PP, WP, LDPE, and RWP have 99.17%, 73.99%, 98.95%, and 81.30% volatile matter, 0.54%, 8.97%, 0.52%, and 5.40% ash content, 0.29%, 6.39%, 0.38%, and 3.20% fixed carbon, respectively. The reported volatile matter for PP, WP, LDPE, and WRP has a good agreement with other reported samples in Table 1. Volatile matter in pyrolysis is crucial as it influences the yield and composition of pyrolysis products. A higher volatile matter typically results in increased production of bio-oil and syngas, while lower volatile matter leads to a higher yield of biochar.^31^ The moisture content was absent in PP and LDPE, whereas WP and RWP contained 10.65% and 10.10%, respectively, making them more suitable as pyrolysis feedstock. Moisture content significantly impacts pyrolysis, affecting energy efficiency and product yield. High moisture levels (>10%) require additional energy units to evaporate water, reducing the thermal efficiency and potentially altering the composition of the resulting biochar, bio-oil, and syngas.^32^ The ash content of PP, WP, LDPE, and WRP is 0.54%, 8.97%, 0.52%, and 5.40%, respectively. The reported ash content matches well with the other reported studies in Table 1. Ash content in pyrolysis impacts the quality and usability of the resulting biochar and pyrolysis oil. A higher ash content (>20–30%) can lower the HHV of pyrolysis oil and increase the ash content in biochar, thereby affecting its suitability as a soil amendment, energy storage material, and carbon sequestration material. Minimising ash content is crucial for maximising the energy potential and application versatility of pyrolysis products.^31,32^ Finally, the fixed carbon content was found to be 0.29%, 6.39%, 0.38%, and 3.20%, respectively, which is in good agreement with other reported biomass values in Table 1. Fixed carbon content in pyrolysis influences the energy density and stability of the resulting biochar and pyrolysis oil. A higher fixed carbon content indicates greater carbon sequestration potential in biochar and improved energy content in pyrolysis oil, thereby enhancing combustion efficiency. This parameter is critical for optimising pyrolysis processes to produce high-quality biofuels and carbon-rich materials for various industrial and environmental applications.^31^ The elemental composition of PP, WP, LDPE, and WRP is reported in Table 1. PP, WP, LDPE, and WRP have 85.10, 60.10, 82.70, and 72.22% volatile matter, 13.38, 3.75, 6.75, and 3.14 hydrogen, 1.52, 34.36, 9.13, and 23.57% oxygen, 0, 1.56, 0.11 and 0.99% nitrogen, 1.06, 0.23, 1.31, and 0.007 sulfur content, respectively. The reported results align well with those of other studies in Table 1. Carbon content is pivotal in pyrolysis, as it determines the energy content and environmental impact of the produced biochar. Higher carbon content enhances the calorific value of bio-oil and increases the stability and carbon sequestration potential of biochar, making them valuable renewable energy sources and effective carbon sinks. Efficient management of carbon content is essential for maximising the sustainability and economic viability of pyrolysis processes.^31,32^ Furthermore, the hydrogen content plays a crucial role in pyrolysis, influencing the composition and properties of the pyrolysis products. A higher hydrogen content generally increases pyrolysis yield, making it a valuable renewable liquid fuel or chemical feedstock. However, excessive hydrogen can also increase water content in pyrolysis oil, thereby affecting its stability and combustion properties. Balancing hydrogen content is essential for optimising the efficiency and quality of pyrolysis products for various industrial and energy applications. The oxygen content in pyrolysis affects the quality and properties of char and pyrolysis oil. Higher oxygen content typically results in lower pyrolysis oil energy density and increased char ash content, which affects combustion efficiency and suitability for various applications. Minimising oxygen content can enhance the HHV of pyrolysis products and improve the stability and carbon sequestration potential of char, making it crucial to manage oxygen levels to optimise pyrolysis outcomes.^31^ Finally, nitrogen and sulfur content in pyrolysis influence the environmental and product quality aspects of char and pyrolysis oil. A higher nitrogen content can lead to the formation of nitrogen oxides (NO_*x*_) during combustion, thereby affecting air quality. Sulfur content affects corrosion and combustion emissions, making it essential to minimise sulfur to achieve cleaner-burning biofuels. Managing nitrogen and sulfur content ensures the sustainability and usability of pyrolysis products for energy generation and environmental applications. The bulk densities of PP, WP, LDPE, and WRP were found to be 138, 246, 123, and 146 kg m^−3^, respectively, which are lower than those of waste nitrile gloves. The difference in bulk densities is attributed to the nature and particle size of the feeds. Bulk density in pyrolysis is critical for determining the handling, transportation, and application of char and pyrolysis oil. A higher bulk density of char indicates better carbon sequestration potential and easier handling for soil application, thereby enhancing its effectiveness as a soil amendment and carbon storage medium. For pyrolysis oil, lower bulk density facilitates storage and transportation, ensuring efficiency in its utilisation as a renewable fuel or chemical feedstock.^30^ The higher heating value of PP, WP, LDPE, and WRP was found to be 47.45, 20.55, 36.16, and 25.31 MJ kg^−1^, respectively. The higher heating value (HHV) in pyrolysis indicates the energy content available in the produced char and pyrolysis oil. It directly influences the efficiency and feasibility of utilising these products as renewable fuels or energy sources. A higher HHV signifies greater energy density, enhancing the economic viability and environmental benefits of pyrolysis by producing fuels with higher calorific value and lower emissions^10,30,33^ Waste paper-plastic waste has nitrogen and sulfur content of 0.99 and 0.07%, respectively, which are mostly of paper origin, *i.e.* cellulose proteins, printing inks and adhesives. These heteroatoms are separated in gaseous (NH_3_, H_2_S, and NO*_x_*), liquid (heterocyclic compounds), and solid (char-retained species) products in the course of pyrolysis.

**Table 1:** Physicochemical characterisation of feeds and comparison with other tested studies

Analysis	Polypropylene	Waste paper	Low-density polyethylene	Residual paper-plastic	Polypropylene^27^	Low-density polyethylene^28^	Waste office paper^29^	Waste nitrile gloves^30^
**Proximate analysis (wt%)**								
Moisture content	0	10.65	0.02	10.10	—	0.67	4.50	0.55
Volatile matter	99.17	73.99	98.95	81.30	99.90	99.76	71.90	64.26
Ash content	0.54	8.97	0.52	5.4	0.01	1.57	12.80	4.57
Fixed carbon	0.29	6.39	0.38	3.20	0.09	1.68	10.80	0.67

**Elemental analysis (wt%)**								
Carbon	85.10	60.10	82.70	72.22	83.10	70.94	34.56	76
Hydrogen	13.38	3.75	6.75	3.14	11.77	10.42	4.30	8.54
Oxygen	1.52	34.36	9.13	23.57		18.53	48.25	9.46
Nitrogen	—	1.56	0.11	0.99	0.14	0.09	—	6
Sulpher	1.06	0.23	1.31	0.07	0.16	—	0.09	—
HHV (MJ kg^−1^)	47.45	20.55	36.16	25.31	—	—	13.60	37.12
Bulk density (kg m^−3^)	138 ± 1.6	246 ± 1.2	123 ± 1.2	146 ± 1.4	—	—	—	426.75 ± 1.3

### 3.2. Thermal profile study of feeds

**Fig. 2:**
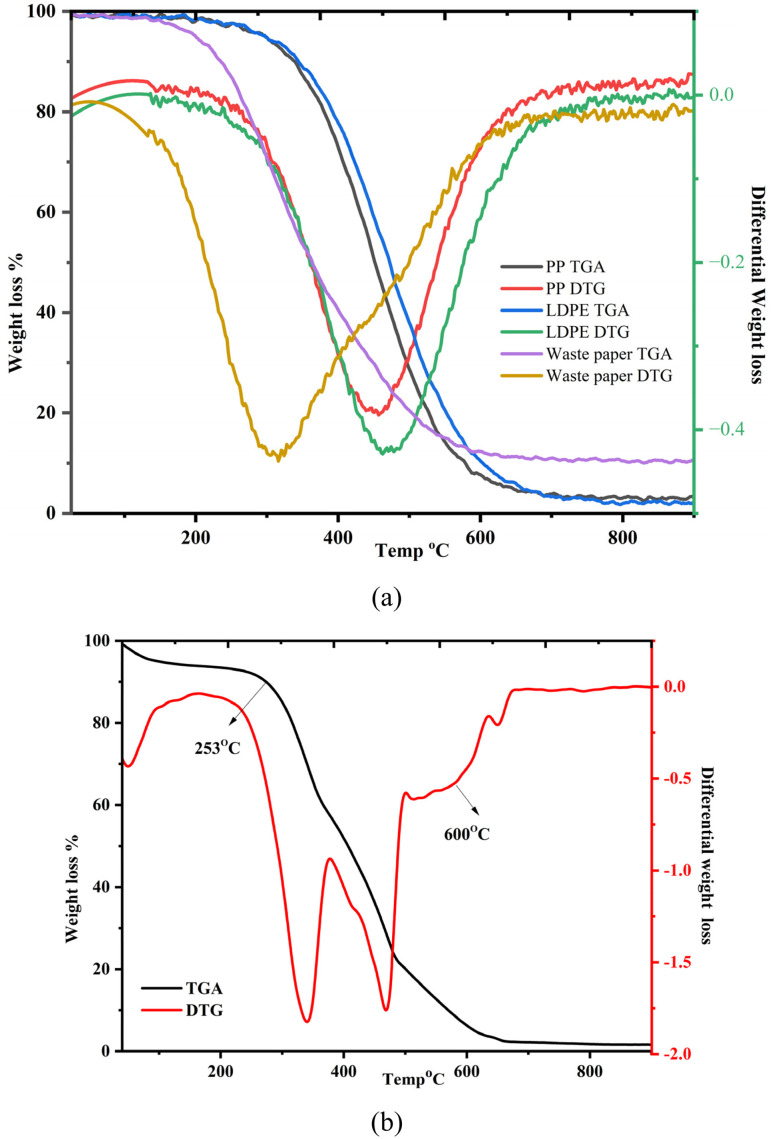
Thermal decomposition profile of (a) PP, LDPE, WP, and (b) paper mill waste.

The thermal profiles of PP, WP, LDPE, and WRP are displayed in Fig. 2a. It was observed that all the feeds decomposed into three main stages: drying, active pyrolysis, and passive pyrolysis. The TGA/DTG curves reveal distinct thermal degradation patterns for PP, LDPE, and waste paper. Polyolefins decompose sharply between 350–500 °C, indicating single-step degradation, whereas waste paper undergoes multi-step loss, with cellulose/hemicellulose degrading at 250–350 °C and lignin beyond 500 °C, resulting in a higher char residue and broader thermal stability. Gradual mass loss observed in the waste paper fraction highlights its inherent oxygenated nature, which is expected to influence the subsequent product distribution by promoting oxygenate formation in the pyrolysis oil fraction. This thermal behaviour, particularly the secondary decomposition beyond 600 °C, forms a crucial basis for understanding the later product yield trends, where increased gas evolution and secondary cracking of volatiles dictate the final distribution of condensable and non-condensable products. Furthermore, Fig. 2b presents the TGA and DTG profiles of the mixed paper-plastic residue, illustrating its thermal decomposition behaviour. The initial weight loss up to 100 °C is attributed to moisture evaporation, a common characteristic of such materials. The degradation then follows two distinct stages. The first major weight loss, occurring between 280 °C and 390 °C, corresponds to the decomposition of cellulose components in paper. Cellulose breaks down rapidly within this range, as expected, leading to a sharp decline in mass. The second degradation phase, from 390 °C to 550 °C, is associated with plastic decomposition, primarily involving PP and LDPE, which are widely used in packaging. The thermal breakdown of these polymers follows the typical behaviour of polyolefins. Interestingly, due to the interaction between paper and plastic, a shift in the decomposition temperature is observed, extending the degradation process up to approximately 650 °C. Furthermore, at 500 °C, around 20% of the material remains as char, and as the temperature increases to 900 °C, the residual char is reduced to less than 5%, indicating minimal inorganic impurities in the sample. Plastics contribute a higher proportion of volatile matter, whereas paper-based materials leave behind more ash due to their inherent inorganic content.^34^ The thermal degradation pattern highlights the complex nature of mixed paper-plastic waste, and the interplay between its components affects its decomposition profile. Understanding these interactions is crucial for optimising thermal conversion processes and improving resource recovery from such heterogeneous waste streams.

### 3.3. Optimisation of temperatures

**Fig. 3:**
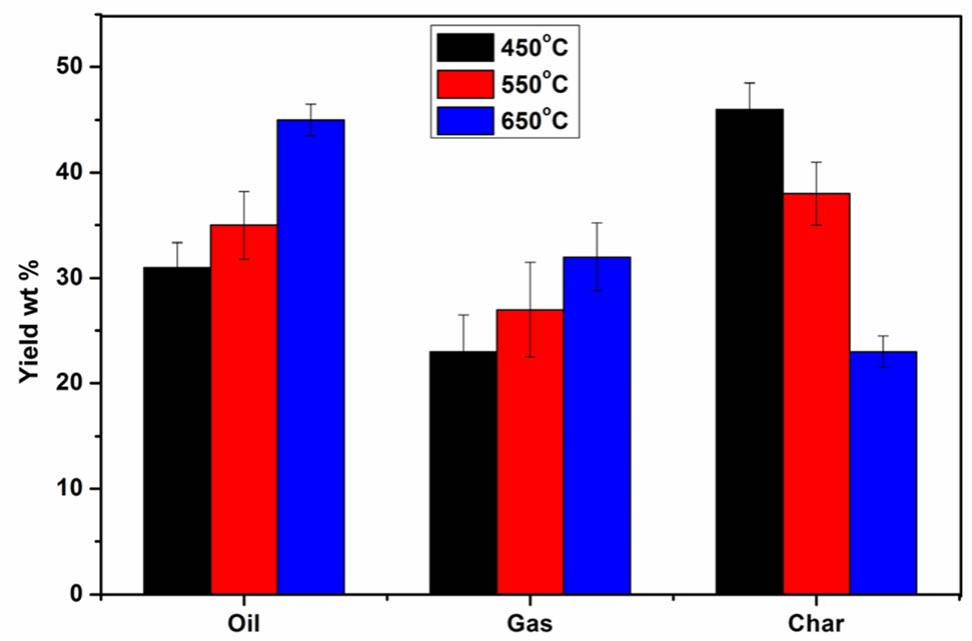
Effect of temperature on the pyrolysis product yield.

The pyrolysis experiment was performed at elevated temperatures (450, 550, and 650 °C), and the pyrolytic product yield is listed in Fig. 3. As the temperature increases, the oil yield rises significantly from approximately 31% at 450 °C to about 45% at 650 °C. This increase is attributed to the enhanced thermal cracking of lignocellulosic biomass components (cellulose, hemicellulose, and lignin), as well as secondary vapour-phase reactions that favour the formation of condensable volatiles, thereby improving bio-oil production.^35,36^ Similarly, the gas yield also shows an upward trend, increasing from around 23% to 32% with rising temperature. This is due to intensified decomposition reactions at higher temperatures, which promote the formation of light, non-condensable gases such as CO, CO_2_, CH_4_, and H_2_.^37^ In contrast, the char yield decreases from about 48% at 450 °C to 24% at 650 °C. The decline in char content results from complete thermal degradation of the biomass, in which higher temperatures convert a greater fraction of the solid material into volatiles. Specifically, a pyrolysis temperature of 650 °C yields the maximum liquid yield and syngas, and a lower char yield due to complete pyrolysis of the feed. The complete pyrolysis refers to the maximum synergistic effect between paper waste and plastic. Furthermore, at 450 and 550 °C, the liquid yield was lower, while the char yield decreased due to incomplete pyrolysis of plastics and paper waste. At lower temperatures (450 °C), the thermal energy is insufficient to break down complex macromolecules effectively, limiting volatile formation.^38^ As the temperature rises, these macromolecules are more thoroughly cracked, producing more condensable vapours that contribute to higher oil yields. Moreover, elevated temperatures can suppress the formation of high-molecular-weight tars, leading to a cleaner, more oil-rich vapour stream.^39^ The syngas yield was also increased from 23% to 32% with temperatures ranging from 450 °C to 650 °C. This trend suggests that higher temperatures favour gasification reactions, including decarboxylation and decarbonylation, which release light gases such as CO_2_, CO, CH_4_, and H_2_. The thermal cracking of vapours and fragments also contributes to gas evolution. At 650 °C, secondary cracking becomes more significant, where even oil vapours and light tars undergo further degradation to form gaseous products, slightly compromising liquid yield but boosting gas production. In contrast, the char yield decreases markedly from 48% to 24% as the pyrolysis temperature increases from 450 to 650 °C. This inverse relationship is expected, as higher temperatures promote complete devolatilization and conversion of the solid biomass matrix. Initially, at 450 °C, a significant portion of the biomass remains unconverted, resulting in a high char yield. However, with increasing temperature (>550 °C), structural breakdown accelerates, and most of the carbonaceous content volatilizes, reducing the formation of solid residue. Additionally, the release of inorganic minerals and the breakdown of cross-linked lignin structures further reduce the stability and mass of char at higher temperatures.^40^ Overall, the data clearly indicate that increasing pyrolysis temperature favours the production of oil and gas while reducing the solid char residue. Demiral and Emine (2011) studied the pyrolysis of grape bagasse at 350–600 °C, heating rate (10–50 °C min^−1^) and nitrogen gas flow rate (50–200 cm^3^ min^−1^). They reported that by increasing the pyrolysis temperature from 350 to 550 °C, the liquid yield increased (maximum pyrolysis oil yield: 27.60%), while the furfural yield decreased at higher temperatures.^35^ Mishra and Mohanty (2020) studied the effect of temperature on waste sawdust and reported that increasing the pyrolysis temperature (400–500 °C) resulted in a 7–8% increase in liquid yield.^38^

### 3.4. Properties of pyrolysis oil

The test revealed that the viscosity of the light pyrolysis oil was low (Table 2). Higher viscosity fuels can cause poor fuel combustion, leading to deposit formation, as well as increased in-cylinder penetration of the fuel spray, which could result in elevated engine pyrolysis oil dilution with fuel. The viscosity at 40 °C (1.3088 cSt) falls within an acceptable range for diesel fuel, indicating good fluidity and pumping characteristics. The density (1000.88 kg m^−3^) was higher than that of typical diesel fuel but still within an acceptable range. Higher density may affect combustion characteristics and engine performance. For commercial LPG and HD-5 quality propane samples, 1a and 1b are considered acceptable, so this test was deemed satisfactory. The flash point (less than 120 °C) met the minimum requirement, indicating potential combustibility. However, it was lower than that of diesel fuel, which may affect safety during storage and transportation. The water content (8.56%) exceeded the maximum limit for diesel fuel, indicating potential contamination or inadequate separation during processing. Excess water can cause engine damage and reduce fuel efficiency. The sulfur content (0.1643%) was below the maximum allowable limit, meeting regulatory requirements for low-sulfur fuels and reducing harmful emissions. Copper strip corrosion was analysed to identify potential issues with copper and bronze fuel system components. The sulfate ash content (0.88%) was within acceptable limits, indicating low levels of inorganic contaminants that could contribute to engine deposits and emissions. Carbon-residue testing was conducted to assess the fuel's tendency to form carbon deposits; the test showed that higher carbon residue was produced. The sediment level (2.56%) exceeds the maximum allowable limit, suggesting inadequate filtration or separation during processing. Excess sediment can cause fuel system clogging and damage to the engine. The absence of inorganic acidity is favourable, indicating good stability and minimal risk of corrosive reactions in the fuel system.

**Table 2:** Properties of pyrolysis oil

Properties	Result	Range/unit	Method of test
Viscosity at 40 °C	1.3088	3.5 to 5.0 cSt	IS 1448 (Part 2S)
Density at 15 °C	1000.88	860–900 kg m^−3^	IS 1448 (Part 16)
Flash point	Less than 120 °C	Min. 101 °C	IS 1448 (Part 21)
Water content	8.56%	Max. 500 mg kg^−1^	IS 1448 (Part 40)
Gross calorific	35–39	MJ kg^−1^	JL/STP/015
Sulfur	0.1643%	Max. 10 mg kg^−1^	IS 1448 (Part 33)
Copper	lb	1a–1b	IS 1448 (Part 151
Sulphate ash	0.88%	Max. 0.02	IS 1448 (Part 4)
Total	1.833%	Max. 24 mg kg^−1^	IP 440
Carbon residue	0.9838%	Max. 0.05%, 5%	IS 1448 (Part 8)
Inorganic	Nil	Nil	IS 1448 (Part 2)
Sediments	2.56%	Max. 0.05%	IS 1448 (Part 30)

### 3.5. Composition analysis of pyrolysis oil

**Fig. 4:**
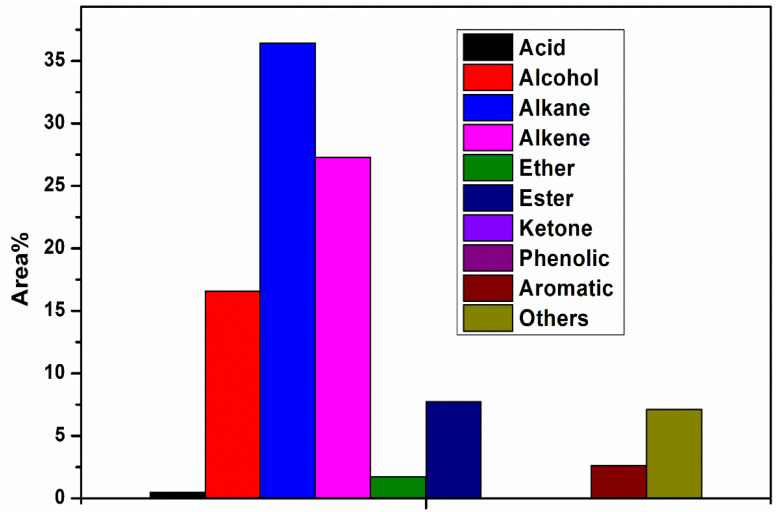
Chemical composition of heavy pyrolysis oil.

Pyrolysis oil produced from pyrolysis contains a diverse range of chemicals with a wide range of molecular weights and different functional groups, including hydroxyl, carbonyl, carboxyl, methyl, and phenolic groups. The condensable fraction collected, using the series condensation system, resulted in two distinct phases: a heavy pyrolysis oil and a light pyrolysis oil. GC-MS analysis revealed that the heavy pyrolysis oil contained higher concentrations of alkanes, alcohols, and alkenes, resulting from the breakage of long-chain hydrocarbon molecules. This fraction was obtained from the first vessel maintained at 200 °C. Molecules with a boiling point lower than 200 °C were condensed in the second vessel. The heavy oil phase contained approximately 35% alkanes, with a smaller fraction of aromatic compounds (Fig. 4). The predominant compounds identified in heavy pyrolysis oil included toluene (3.5%), 2,4 dimethyl-1 heptene (8.95%), ethylbenzene (2.11%), O-xylene (2.08%), styrene (2.60%), *etc.* whereas, light pyrolysis oil exhibited a higher concentration of acids, alcohols, and aromatics, which include 2, isopropyl −5 methyl-1 heptanol (4.45%), 1-heptanol, 2, 4-diethyl (7.13 area%), 1-dodecanol-2 hexyle (3.77 area%). Additionally, alkanes and alkenes were found in nearly equal proportions (Fig. 4). Apart from this, small concentrations of amine, amides, and some unidentified compounds were also detected. Higher amounts of alcohols were produced in the heavy and light pyrolysis oils, which can be attributed to the thermal degradation of cellulose, leading to the formation of hydroxyl functional groups. These hydroxyl groups react with vinyl radicals produced by the cleavage of polyethylene chains, leading to alcohol formation.^41,42^

**Fig. 5:**
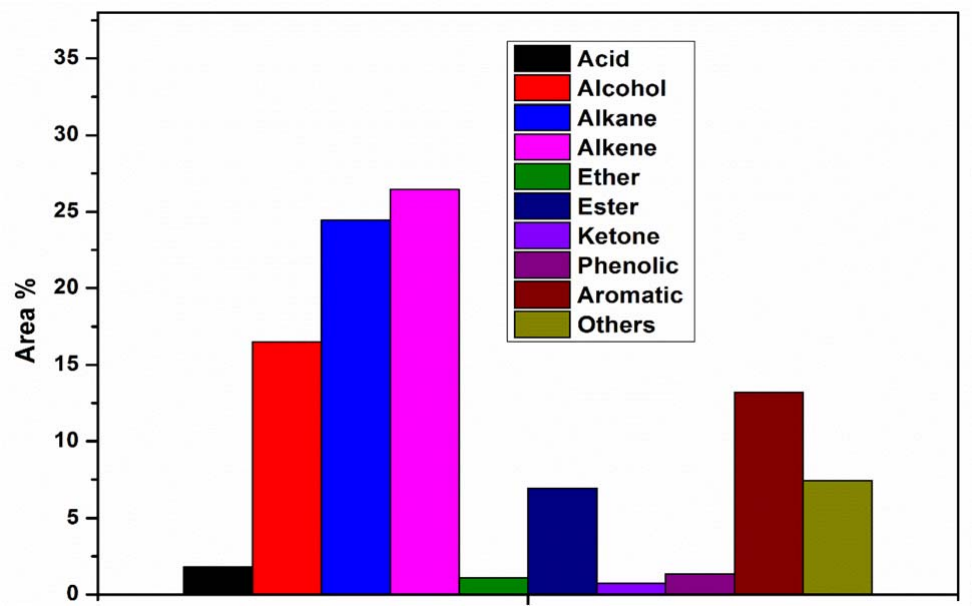
Chemical composition of light pyrolysis oil.

Fig. 5 presents the compositional distribution of pyrolysis oil into major chemical groups based on GC-MS area percentages, clearly indicating that alkenes (27%) and alkanes (24%) dominate the product mixture, reflecting a strong prevalence of hydrocarbon compounds typical of thermally cracked polymeric and lignocellulosic fractions. Alcohols constitute the third-largest group, at 16%, suggesting partial depolymerisation and dehydration reactions of cellulose and hemicellulose components from the biomass feed.^43,44^ Meanwhile, aromatics (13%) and phenolic compounds (8%) appear in notable quantities, consistent with aromatic ring formation, lignin decomposition, and secondary aromatisation pathways. Ketones (3%) and esters (7%) represent minor but significant oxygenated species formed through rearrangements, fragmentation, and esterification reactions of carbohydrates, whereas acids (2%) and ethers (1%) are present only in trace amounts, indicating efficient decarboxylation, deoxygenation, and cracking processes that minimise corrosive or unstable acidic and etheric fractions.^44^ The “others” category (10%) captures miscellaneous low-concentration components likely arising from complex recombination, cyclisation, and secondary cracking reactions during pyrolysis. The distribution pattern reflects an effective thermal conversion pathway that favours hydrocarbon-rich oil with relatively lower oxygenated compound content, suggesting improved fuel quality, a higher calorific value, and a reduced need for post-treatment compared to conventional bio-oil derived solely from biomass. The dominance of alkanes and alkenes highlights the prevalence of strong cracking and hydrogen transfer reactions, which are potentially facilitated by co-pyrolysis interactions when plastics are present, thereby enhancing hydrocarbon formation and suppressing the persistence of oxygenated molecules.^45^ The concurrent presence of aromatics and phenolics indicates the partial operation of lignin-derived pathways and hydrocarbon recombination, leading to aromatic structures that contribute to energy density but may require stabilisation to prevent polymerisation during storage. This compositional profile highlights the potential of optimised pyrolysis to produce liquid fuels rich in valuable hydrocarbon species, suitable for energy and chemical recovery.

**Fig. 6:**
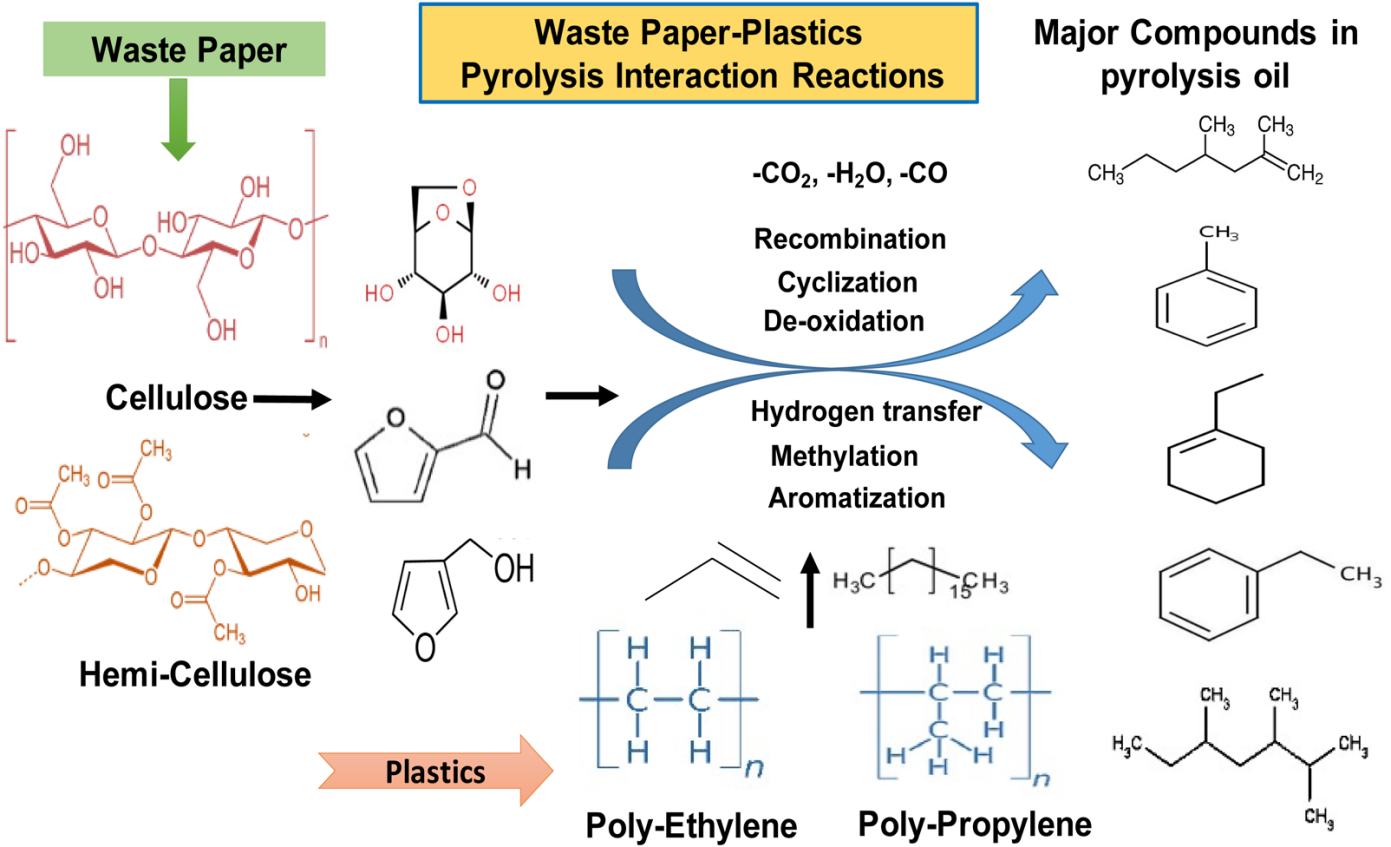
Reaction pathway of co-pyrolysis of biomass and plastics.

The thermal decomposition pathway of plastic-biomass co-pyrolysis predominantly follows a free radical degradation and recombination mechanism. In thermal cracking, the pathway involves random chain scission, chain-end scission, or the removal of pendant groups. These degradation pathways contribute to higher concentrations of branched aliphatic compounds in the pyrolysis oil. Fig. 6 displays the sequence that illustrates the synergistic pyrolysis interaction mechanisms occurring during the co-conversion of waste paper and plastic polymers, demonstrating cellulose and hemicellulose from paper and polyethylene (PE) and polypropylene (PP) from plastics undergo thermal degradation and subsequent reactive transformations to yield high-value hydrocarbons in the pyrolysis oil. Waste paper contains polysaccharides, such as cellulose and hemicellulose, that thermally decompose into oxygenated intermediates, including furans and levoglucosan-like compounds. These compounds typically release significant amounts of CO_2_, H_2_O, and CO due to deoxygenation reactions.^46–48^ In contrast, plastics primarily consist of long-chain hydrocarbons lacking oxygen, which crack into olefins and paraffins under heat.^49^ The interaction between lignocellulosic and plastic-derived radicals enhances hydrogen transfer, thereby improving the stability of reactive intermediates and reducing the oxygen content in the product oil, which in turn increases the hydrocarbon yield compared to individual feedstock pyrolysis. Key secondary reactions, such as recombination, cyclisation, de-oxidation, methylation and aromatisation, facilitate the formation of major compounds, including alkanes, alkenes, benzene derivatives, and substituted aromatic hydrocarbons.^49^ This synergistic pathway helps overcome the challenge of high oxygen content in biomass pyrolysis oil, thereby improving its fuel characteristics. Simultaneously, plastics supply hydrogen to stabilise biomass-derived free radicals and suppress char formation, while biomass oxygen promotes the cracking of polymer chains and increases the formation of light hydrocarbons.^46–48^ As a result, mixed feed pyrolysis typically produces more deoxygenated, energy-dense liquid fuels with lower acidity and a higher calorific value than single-component pyrolysis, reflecting the beneficial effects of material co-processing.^46–48^Fig. 5 highlights that the waste paper-plastic co-pyrolysis leverages complementary chemical pathways, such as oxygen removal from biomass fragments and hydrogen donation from plastics, to generate value-added aromatic and aliphatic hydrocarbons while minimising waste, thus offering a sustainable pathway for integrated solid waste conversion into cleaner biofuel intermediates. The chemical composition provides certain transformation mechanisms during co-pyrolysis. The high percentage of alkenes in light oil (Fig. 5) indicates that the radicals derived from plastics are scissored at low molecular weights. Coexistence of alcohols (approximately 16–17% area) and alkanes (approximately 36% in Fig. 4, 24% in Fig. 5) indicates that the saturated plastic chains transferred hydrogen to cellulose-derived oxygenates, inhibiting their oxygen functionality. Distribution of aromatic compounds differs: phenolics (2.5% in Fig. 4, 13% in Fig. 5) are formed mainly when lignin is depolymerised, whereas non-phenolic aromatics are produced by cellulose fragments and plastic-derived dienes *via* Diels–Alder cyclisation. The content of the esters (7.5% in Fig. 4, 6.8% in Fig. 5) shows the fact that in the process of the co-processing, cellulosic intermediates were not entirely deoxygenated. Quantitative comparison between mixed-feedstock pyrolysis and individual feedstock shows synergy: the alkane yield is 8–12% higher than expected based on the weighted average, and oxygen-containing compounds are also reduced by 15% as a result of deoxygenation driven by radical coupling between plastic and biomass decomposition intermediates. Fig. 6. Reaction pathways suggested in the co-pyrolysis of waste papers and plastic. Major pathways include cellulose depolymerisation *via* glycosidic breakage to furan intermediates, plastic chain scission to olefinic radicals (C_4_–C_12_ alkenes, 26–27% of products), along with oxygen transfer of plastic-derived radicals to biomass oxygenates and aromatisation *via* cyclisation-dehydrogenation.

### 3.6. Fractional upgradation of pyrolysis oil using vacuum evaporation

**Fig. 7:**
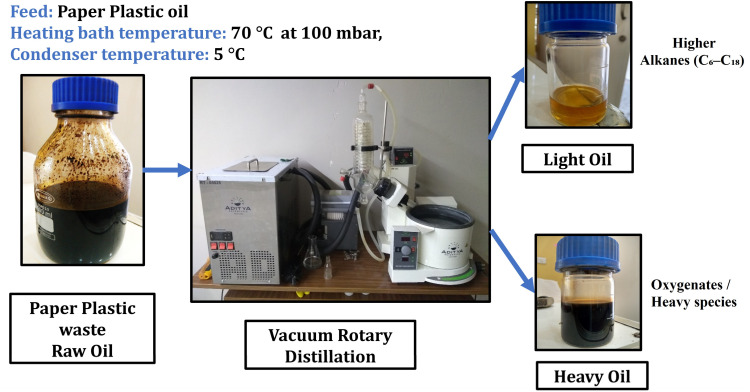
Vacuum rotary evaporation studies.

The heavy oil contained polymerised solids and impurities, necessitating further purification. Rotary evaporation was employed as a low-energy, low-cost pre-fractionation step to separate light and heavy bio-oil fractions based on volatility. Therefore, vacuum rotary distillation was employed to fractionate the pyrolysis oil into lighter and heavier fractions suitable for improved utilisation and application. By combining low pressure with a moderate bath temperature, the process selectively enriches the light fraction in fuel-range hydrocarbons (C_6_–C_18_) while leaving behind heavy, high-boiling species. The distillation efficiency was optimised by maintaining 100 mbar pressure and 70 °C bath temperature, which selectively volatilized C_6_–C_18_ hydrocarbons while suppressing thermal degradation. The condenser at 5 °C ensured effective recovery of light fractions, thereby justifying the claim of upgraded fuel-range hydrocarbons (Fig. 7). The heavy pyrolysis oil was pre-filtered before distillation. Whatman filter paper removed approximately 10 wt% of the solid residue, allowing 90% of the distillate to be processed through the vacuum rotary distiller. Table 3 presents a detailed breakdown of the major chemical constituents in heavier and lighter fractions. During vacuum distillation, at 100 mbar pressure, approximately 15% lighter compounds were separated while 85% heavier fractions remained in the rotary flask. GC-MS analysis revealed that the lighter fraction primarily contained 80% hydrocarbons in the C_6_–C_12_ range, with smaller portions in the C_12_–C_18_ range and traces up to C_18_–C_30_. The heavier fraction was predominantly composed of C_9_–C_18_ hydrocarbons, with minor high-molecular-weight compounds extending up to C_38_. Oxygenated compounds were found in higher concentrations in the heavier fraction. Furthermore, oxygenated compounds were found in higher concentrations in the heavier fraction, likely due to thermal degradation products of cellulose-based materials.^50^

**Table 3:** Major compounds in heavy and light pyrolysis oil from the vacuum rotary evaporator

Hydrocarbon class (C_6_–C_18_)	Light oil fraction	Heavy oil fraction	Interpretation
Alkanes (C_6_–C_18_)	Abundant (heptane, octane, nonane, decane, undecane)	Moderate presence	Light fraction enriched in volatile straight-chain hydrocarbons
Alkenes (C_6_–C_18_)	Significant (1-octene, 2-octene, 2,4-dimethyl-1-heptene, 1-undecene)	Moderate	Light fraction shows more low-boiling olefins, indicating upgrading
Aromatics (C_6_–C_18_)	Strong (toluene, ethylbenzene, xylenes, mesitylene)	Present but less diverse	Light fraction enriched in fuel-relevant aromatics
Cyclic hydrocarbons	Moderate (cyclohexane, cyclopentane derivatives)	Similar distribution	Both fractions contain cyclics, but lighter ones dominate in light oil
Oxygenates/heavy species	Trace (<2%)	Significant (>20%, *e.g.* neophytadiene, esters, siloxanes)	Heavy fraction retains non-fuel oxygenates and high-MW compounds

The pyrolysis oil derived from paper mill waste exhibits several fuel properties comparable to those of diesel, including viscosity, calorific value, sulfur content, and copper strip corrosion behaviour.^43^ These characteristics highlight its potential as a renewable fuel substitute. However, certain limitations, such as higher water and sediment content, must be addressed to ensure reliable performance and compatibility with diesel engines. Additionally, the relatively lower flash point and higher density of the pyrolysis oil may require modifications in fuel handling and engine calibration to ensure safe and efficient operation.^50^ The feedstock received from paper recycling facilities typically contains a significant proportion of plastics, as most recoverable paper fibres have already been extracted. Consequently, the quality and composition of the resulting pyrolysis oil are strongly influenced by the plastic content in the waste. Heavy pyrolysis oil is dominated by long-chain hydrocarbons, whereas light pyrolysis oil contains more oxygenated compounds such as ketones, phenols, and organic acids.^51,52^ The gross calorific value of the produced oil was comparable to conventional diesel, confirming its strong energy potential for combustion applications. Specifically, heavy pyrolysis oil exhibited a calorific value of 40–43 MJ kg^−1^, while light pyrolysis oil ranged from 36–39 MJ kg^−1^. The char fraction also possessed a considerable calorific value of 20–22 MJ kg^−1^, making it suitable for potential energy recovery or solid fuel applications.^53^ The pyrolysis oil from paper mill waste shows considerable promise as a sustainable alternative to diesel fuel; further refinement and optimisation of the fuel properties are essential to meet stringent performance and quality standards.

### 3.7. Characterisation of char

The pyrolysis of paper-plastic waste generated from paper mill recycling streams produced oil, gas, and solid char with characteristics demonstrating strong energy recovery potential, although additional refinement is needed for filler grade applications. The proximate and ultimate analysis of the char revealed 0.73% loss on drying (LOD), 52.16% volatile matter, 16.8% fixed carbon, and 30.30% ash, accompanied by elemental composition of 71.2% carbon, 2.17% hydrogen, 0.99% nitrogen, 0.07% sulfur, and 25.56% oxygen, with an energy content of 3945 cal g^−1^ (16.5 MJ kg^−1^), indicating use as alternative secondary solid fuel or precursor for activated carbon production (Table 4). The proximate and ultimate analysis results indicate that the char produced from paper-plastic pyrolysis has valuable fuel and material properties.^54^ The high carbon content (71.20%) and moderate fixed-carbon fraction (16.8%) indicate a strong energy storage potential.^53^ The volatile matter (52.16%) supports good ignition behaviour, helping the char burn more easily, although it may also require stabilisation if used for activated carbon production. The char can serve as secondary solid fuel with an energy value of 16.50 MJ kg^−1^, slightly lower than typical coal (20–30 MJ kg^−1^). Its composition makes it a promising raw material for producing activated carbon for filtration or adsorption applications.^55^ The very low sulfur (0.07%) and nitrogen (0.99%) contents imply that emissions of SO_*x*_ and NO_*x*_ during combustion would be minimal, making the char environmentally favourable.^55–57^ Additionally reduced SO_*x*_ and NO_*x*_ emissions, and sustainable thermal conversion performance, makes the char as a viable substitute fuel for industrial heating systems and gasification processes.^58–60^

**Table 4:** Ultimate and proximate analysis of char

Compounds wt%	LOD	Ash	Volatile matter	Fix carbon	Carbon	Hydrogen	Nitrogen	Sulfur	Oxygen	Calorific value (cal per gm)
Paper-plastic char	0.73	30.3	52.16	16.8	71.2	2.17	0.99	0.07	25.56	3945

### 3.8. XRF analysis of ash

**Table 5:** XRF analysis of paper-plastic char

Compound	MgO	Al_2_O_3_	SiO_2_	P_2_O_5_	SO_3_	K_2_O	CaO	TiO_2_	Fe_2_O_3_	In_2_O_3_	Cl	Ignition loss
Weight %	0.67	1.95	3.3	0.12	0.86	0.47	29.7	2.6	3.68	0.85	5.55	49.88

The XRF (Table 5) results have indicated that the char is made of paper-plastic mixture, which consists of considerable mineral (CaO constitutes 29.7 wt%), Fe_2_O_3_, TiO_2_, and SiO_2_, with minor components of MgO, Al_2_O_3_, K_2_O, and SO_3_. Inherent alkalinity and possible reinforcing behaviour under inclusion in the polymer matrices have been indicated by the high CaO content, and Fe_2_O_3_ and TiO_2_ which increases the thermal stability, UV stability and mechanical reinforcement of materials. The presence of carbonaceous material has been proved to be predominant by the relatively high ignition loss (49.88) and char is suited to be mineral-carbon hybrid filler and simultaneously energy-bearing material. The presence of transition‑metal and alkaline oxide phases suggests potential catalytic activity, as they provide active surface sites that facilitate oxidation and gas–solid reactions These compositional properties have depicted that the char is not just a byproduct of pyrolysis, but a viable inorganic-carbon composite which could be the source for value-added materials. presence of transition-metal and alkaline oxide phases has indicated potential ^61,62^

### 3.9. SEM/EDS analysis

**Fig. 8:**
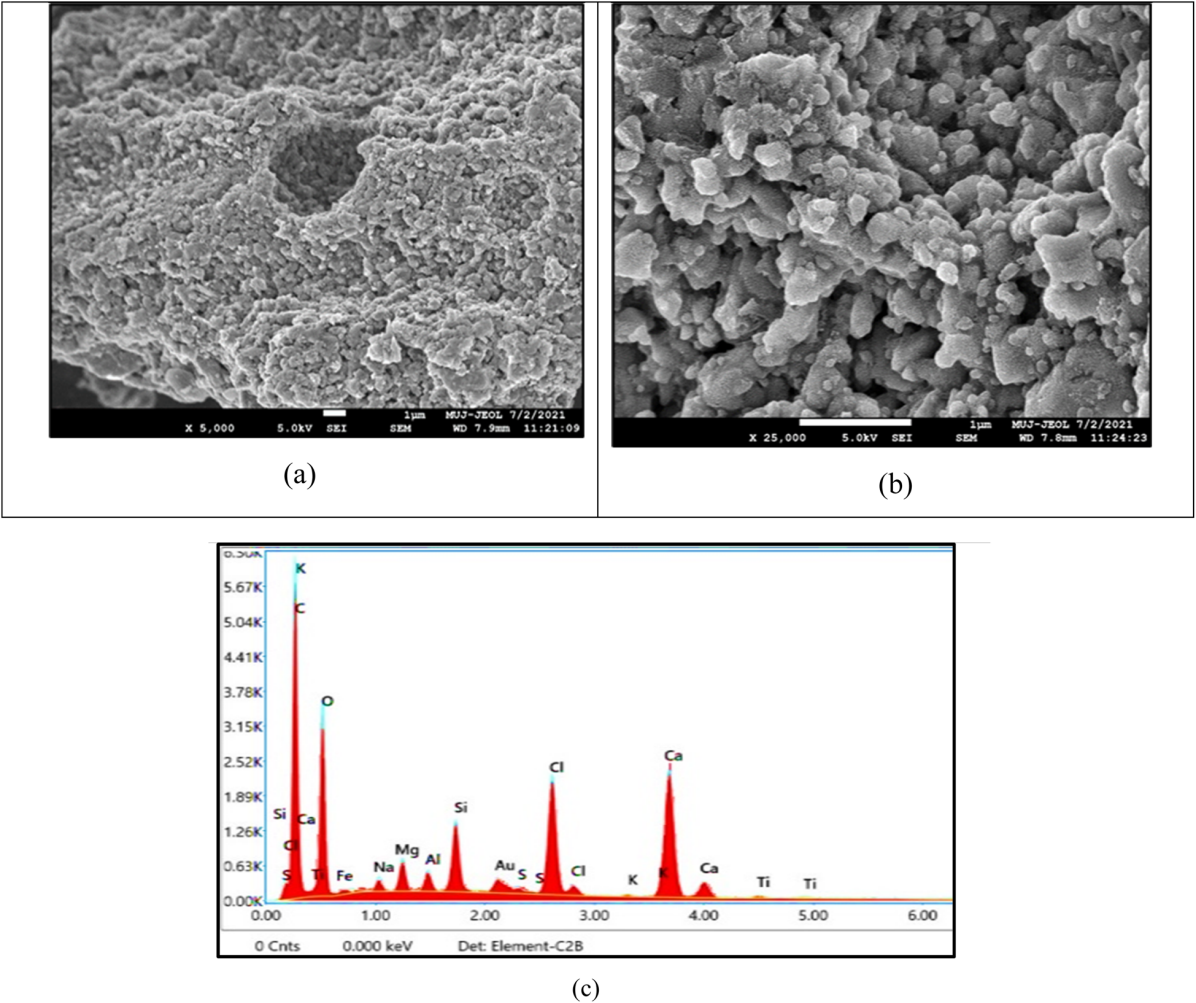
(a) and (b) SEM images of the pyrolysis char of the paper-plastic mixture at 5 and 25 KX and (c) EDS/Elemental composition of the pyrolysis char.

The SEM micrographs of the produced char clearly reveal a heterogeneous, rough, and highly porous morphology, as shown in Fig. 8a and b. SEM results support the physicochemical and energetic findings from proximate, ultimate, and XRF analyses, and confirm its suitability as secondary solid fuel and activated carbon precursors. At 5000× magnification, the surface displays irregular pore structures and loosely bonded particulate layers, indicative of volatile release during pyrolysis, which contributes to an enhanced surface area beneficial for adsorption, catalyst support, and gas-solid reaction processes (Fig. 8a). The 250 00× magnified image further highlights clusters of fine micro-granular particles and interconnected micro-pores, suggesting partially developed porosity and carbonaceous matrix evolution essential for activation processes (Fig. 8b). These morphological features align with proximate analysis, reflecting significant devolatilization and low inorganic residue, which supports easier pore development during activation and enhances combustion efficiency.^63^ The presence of micro-textured carbon clusters observed in SEM suggests favourable internal bonding and structural integrity, enabling high reactivity in thermal systems and ease of surface modification for adsorption applications.^64^ The granular and porous nature of the char indicates that during pyrolysis, significant decomposition of cellulose, hemicellulose, lignin, and plastics occurred, creating active sites that are beneficial for capturing pollutants, adsorbing organic molecules, or supporting catalytic nanoparticles.^33^ These morphological and compositional characteristics emphasise that char derived from paper-plastic waste is not merely a by-product but a value-added material with multifunctional potential: as a cleaner solid fuel alternative to low-grade coal, as a precursor for activated carbon in water and gas purification, and as a low-cost catalyst support in industrial and environmental processes.^65^ The SEM results, coupled with chemical and thermal data, confirm that this waste-derived char is structurally advantageous, chemically enriched, and environmentally relevant, supporting circular-economy objectives by transforming industrial paper and plastic residues into high-value carbonaceous products rather than a disposal burden. EDS results (Fig. 8c) further validate its composition with 42.4% carbon and 28.5% oxygen, along with minerals such as Ca (13.5%), Si (2.6%), Mg (1.4%), and Fe (0.7%), which impart catalytic and adsorption benefits while maintaining low sulfur (0.3%) and chlorine (7.1%) contents (Table 6). The porous carbon matrix and mineral content significantly enhance its suitability for environmental applications, including wastewater treatment, gas purification, and catalyst support.^66^ This demonstrates that waste-derived char has functional value beyond fuel, supporting the circular economy's resource recovery.

**Table 6:** EDS analysis of paper-plastic char

Element	C	O	Na	Mg	Al	Si	S	Cl	K	Ca	Ti	Fe
Weight %	42.4	28.5	0.9	1.4	0.8	2.6	0.3	7.1	0.2	13.5	0.4	0.7

### 3.10. Char for rubber compounding

The compounding study compared three SBR-1502 rubber formulations standard carbon-black-filled (STD), char-filled, and blank each containing consistent quantities of SBR, ZnO, stearic acid, accelerators, and sulfur, but differing in filler type: the STD contained 50 phr N774 carbon black, the char formulation contained 50 phr pyrolysis char, and the blank had no filler (Table 7). The purpose of this design was to evaluate pyrolysis-char as a sustainable reinforcing alternative to conventional carbon black. The carbon-black-filled standard generally represents an optimal reinforcement, as it improves tensile strength, modulus, abrasion resistance, and hardness due to its controlled particle size, structure, and strong interfacial interaction with rubber.^67^ In contrast, the char formulation substitutes bio-origin pyrolytic char, typically characterised by a broader particle-size distribution, more ash, and less uniform surface chemistry factors that influence reinforcement intensity.^68^ Moreover, the expected trends include intermediate performance for the char-filled compound, with mechanical strength and hardness likely higher than those of the blank (due to filler-rubber interaction and increased surface crosslinking), but lower than the standard due to lower dispersion efficiency and filler purity.^69^ The blank compound, containing no reinforcing filler, is anticipated to show the lowest performance with higher elongation and lower hardness, demonstrating the necessity of filler reinforcement in elastomers. The flow improvements in char vulcanizates may result from a porous surface morphology that enhances plasticity at processing temperatures, while the higher accelerator (TBBS) in the blank indicates compensation for the absence of filler activation.^70^ The study explores waste-derived char as a circular economy filler strategy, where adequate reinforcement, combined with cost and sustainability benefits, could justify the partial replacement of carbon black. The formulation comparison provides foundational evidence on reinforcing capability, processing behaviour, and cure system response, highlighting the green potential and performance limitations of pyrolysis-char-reinforced elastomers relative to conventional carbon black.^71^

**Table 7:** Compounding ingredient of char

Ingredients	STD	Char	Blank
	Ph^a^	Phr^a^	Phr^a^
SBR1502	100	100	100
ZnO	3	3	3
SA	2	2	2
Carbon black (N774)	50	0	0
Char	0	50	0
CBS	1.5	1.5	1.5
TBBS	0.5	0.5	1.5
SULPHUR	1.5	1.5	1.5

a Parts per hundred rubber.

The rheometric and moulding results clearly demonstrate the differences in reinforcing efficiency and curing behaviour between the standard carbon-black-filled SBR compound and the char-filled compound (Table 8). The standard (STD) formulation exhibits significantly higher maximum torque (MH = 14.19 dN m) compared to the char compound (MH = 8.9 dN m), indicating stronger crosslinking density and reinforcement intensity, which is expected because commercial carbon black (N774) offers superior surface activity and interaction with rubber chains. Meanwhile, the char-filled compound exhibits a higher minimum torque (ML), reflecting greater viscosity during the early stages of cure, likely due to the char's complex morphology and presence of inorganic residues.^72^ Faster cure behavior is observed in the char sample, with shorter scorch time (*T*_S2_ = 0.83 min) and reduced optimal cure time (*T*_c90_ = 9.11 min) compared to the STD sample (*T*_S2_ = 1.11 min and *T*_c90_ = 11.86 min), which indicates char's catalytic effect on vulcanization, possibly attributed to metal traces or residual ash acting as cure activator components. This is further supported by the observation of metallic traces in the uncured char batch. The reduced moulding time required at 160 °C for the char compound (11 min) compared to the standard (STD, 13 min) emphasises improved cure kinetics and enhanced processing efficiency. However, the lower torque values of the char sample also suggest that although char accelerates the curing process, it does not reinforce the rubber matrix as effectively as standard carbon black.^67^ Overall, the results highlight a trade-off: pyrolysis char offers faster curing and reduced moulding time due to its reactive surface constituents, but provides moderate reinforcement compared to conventional carbon black. This demonstrates char's potential as a partial sustainable filler replacement to reduce cost and support circular waste-to-material concepts, particularly in applications where intermediate mechanical properties are acceptable.

**Table 8:** Rheometric properties of rubber compound and its moulding torque testing of char

Properties tested	STD	Char
Maximum torque (MH)	14.19	8.9
Minimum torque (ML)	1.11	0.83
*T*_S2_ (min)	4.38	4.07
*T*_c90_ (min)	11.86	9.11
Molding time for slab@160 °C, min	13	11
Uncured batch	OK	Metallic traces

The comparative mechanical and rheometric analysis between the standard carbon-black-reinforced rubber compound, the char-filled compound, and the blank formulation clearly highlights the reinforcing and curing characteristics of pyrolysis char (Table 9). The standard (STD) compound demonstrated the strongest mechanical properties, with hardness of 63–64 Shore A, tensile strength of 123 kg cm^−2^, modulus at 300% of 113 kg cm^−2^, and tear strength of 66 kg cm^−1^, confirming the superior reinforcing ability of commercial carbon black due to its high surface activity, optimized particle size, and strong rubber-filler compatibility.^73^ In contrast, the char-filled compound exhibited moderate reinforcement, with a hardness of 58 Shore A, a tensile strength of 18 kg cm^−2^, a modulus of 300% of 18 kg cm^−2^, and a tear strength of 15 kg cm^−1^. These reductions can be attributed to char's heterogeneous particle distribution, higher ash content, and less-active surface chemistry, which reduce its interaction with the rubber matrix compared to well-structured carbon black.^73^ However, the char compound still performed significantly better than the blank formulation, which showed the lowest hardness (46 Shore A), modulus (16 kg cm^−2^ at 300%), and tear strength (14 kg cm^−1^). This emphasises the essential role of fillers in elastomer reinforcement. Interestingly, elongation at break followed an opposite trend, where the blank exhibited the highest elongation (350%), followed by the char (322%) and STD (320%), demonstrating that lower reinforcement enables greater elasticity. Coupled with earlier rheometric results showing reduced cure time (*T*_c90_ = 9.11 min for char *vs.* 11.86 min for STD) due to the catalytic effect of residual metals and ash, the findings indicate that char enhances curing efficiency but provides lower reinforcement strength.^67^ This performance balance makes pyrolysis char suitable for cost-effective and sustainable rubber applications where moderate mechanical properties and faster processing are advantageous. The results confirm that while char cannot fully replace carbon black in high-performance applications, it holds strong potential as a partial or economical filler, supporting circular waste-to-resource utilisation by transforming pyrolysis by-products into value-added reinforcing agents.^67,69,72^ The mechanical testing results show char's viability as a rubber filler, though the lower reinforcement compared to N774 suggests that char properties optimisations, particularly surface area, particle structure, through activation or modification processes, could significantly enhance its performance to approach commercial carbon black standards. Although the 85% reduction in tensile strength limits high-performance uses, char-filled rubber meets ASTM D2000-M and ISO 2230 Grade 4 specifications for non-load-bearing seals and gaskets. The 35–42% cost reduction and faster cure time (*T*_c90_, 9.11 min) position char competitively in low-specification rubber markets where moderate mechanical properties and processing efficiency are prioritized over maximum performance.

**Table 9:** Modulus and hardness testing of char

Properties tested	Test method	STD	Char	Blank
Hardness (Shore A)	ASTM D2240	63/64	58	46
Modulus at 100% (kg cm)^−2^	ASTM D412	23	12	9
Modulus at 200% (kg cm)^−2^		61	15	—
Modulus at 300% (kg cm)^−2^		113	18	16
Tensile strength (kg cm)^−2^		123	18	18
Elongation at break (%)		320	322	350
Tear strength (kg cm^−1^)	ASTM D624	66	15	14

## 4 Conclusions and future study recommendations

This study successfully demonstrated a sustainable, scalable, and circular-economy-driven approach for upcycling paper-mill residual waste by co-pyrolyzing mixed paper-plastic streams in a semi-pilot-scale rotary kiln reactor. The optimised pyrolysis temperature range of 450–650 °C achieved a maximum liquid fuel yield of 48 wt%, along with 20 wt% char and syngas, confirming efficient thermal conversion and synergistic interaction between lignocellulosic and polymeric components. The produced pyrolysis oil exhibited high calorific value (up to 43 MJ kg^−1^), significant hydrocarbon dominance, and low sulfur content, making it a promising alternative renewable fuel after minimal upgrading. Vacuum distillation further enhanced oil quality, concentrating C_6_–C_18_ fractions suitable for fuel and chemical recovery. Furthermore, the solid char residue exhibited a desirable carbon-rich composition, porous morphology, indicating its potential for energy applications and advanced material use (adsorbent, catalytic support, activated carbon precursor). Rubber compounding trials revealed that char could partially substitute carbon black, demonstrating faster cure kinetics and acceptable mechanical performance for industrial applications where moderate strength and flexibility are sufficient. Overall, this research provides strong evidence that the pyrolytic conversion of paper-mill waste is technically feasible and aligns with sustainable waste valorisation strategies, pollution prevention goals, and industrial material recovery pathways. The study advances an integrated framework for resource-efficient utilisation of solid paper-plastic residues, contributing to circular manufacturing and reduced landfill burden.

To accelerate industrial implementation and enhance performance outcomes, several future directions are recommended. First, extensive post-processing and upgrading of pyrolysis oil, such as hydrotreating, catalytic deoxygenation, or membrane-based purification, should be explored to improve fuel stability, reduce oxygen and water content, and achieve diesel-standard quality. Blending studies with diesel and commercial biodiesel under engine evaluation would provide real-world performance validation. For char valorisation, surface modification strategies such as activation (steam/chemical), acid washing, or nano-functionalization should be pursued to enhance specific surface area, reduce impurities, and improve compatibility in polymer composites. Addressing the removal of metal and inorganic trace contaminants through magnetic separation or flotation will enable its use in high-purity sectors, including electronics, battery carbon, and water treatment. Additionally, comparative life-cycle assessment and techno-economic analysis should be performed to establish cost competitiveness, environmental benefits, and energy payback. The optimisation of the process through continuous-mode pyrolysis, catalyst integration, reactor design improvements, and automated feed handling will be critical for full-scale commercialisation. AI-enabled process control and predictive modelling may further increase product selectivity and energy efficiency. Scaling studies in industrial settings, particularly within paper mill clusters, can support decentralised waste-to-fuel production hubs, reducing logistics and promoting local resource circularity. Furthermore, policy support, including incentives for waste valorisation, carbon credit mechanisms, and industrial symbiosis frameworks, will facilitate adoption. Collaboration with the tyre, rubber, petrochemical, and energy industries will expand the utilisation pathways for products. Future work should integrate advanced upgrading, performance benchmarking, system-scale modelling, and industry partnership development to realise the full techno-economic viability and environmental sustainability of paper-mill waste pyrolysis.

## Ethical statement

This work does not contain any research involving humans or animals.

## Conflicts of interest

The authors declare that they have no relevant financial or non-financial interests to disclose that could have appeared to influence the work reported in this paper.

## Data Availability

The datasets generated during and/or analysed during the current study are available from the corresponding author upon reasonable request.
